# Norethisterone‐Associated Decidual Cast Expulsion Mimicking an Acute Gynecologic Emergency: A Case Report

**DOI:** 10.1002/ccr3.73549

**Published:** 2026-09-15

**Authors:** Tuka Hamasho, Nazaha Al‐hassoun, Nagham Al‐hassoun, Mohammad Al Diab Al Azzawi, Hiba Abdullah, Walid al saleh

**Affiliations:** ^1^ Faculty of Medicine Hama University Hama Syrian Arab Republic; ^2^ Faculty of Medicine The National Ribat University Khartoum Sudan; ^3^ Department of Obstetrics and Gynecology Qarah Hospital Damascus Syria; ^4^ Department of Pathology Damascus University Obstetrics Hospital Damascus Syria

**Keywords:** case report, decidual cast, membranous dysmenorrhea, norethisterone

## Abstract

Decidual cast is a rare benign condition that may mimic acute gynecologic emergencies. It should be suspected in patients with severe colicky pain followed by sudden relief after tissue expulsion, especially with progestin use such as norethisterone. Accurate diagnosis and histopathology prevent unnecessary surgery.

## 1 Introduction

Endometrial cast, also known as membranous dysmenorrhea or decidual cast, is a rare benign condition characterized by the spontaneous sloughing of the entire endometrium as a single piece that retains the shape of the uterine cavity [1, 2, 3, 4]. Only a limited number of cases have been reported in the literature, with the majority occurring in adolescents [2]. The exact pathophysiology remains unclear, and it has been termed “the disease of theories” [5]. Proposed mechanisms include hormonal imbalance with incomplete desquamation of the thickened endometrium, vascular dysfunction involving spiral artery vasospasm, and altered cell adhesion mediated by integrins [3, 5]. A decidual reaction occurs as an exaggerated endometrial response to elevated progesterone levels, whether endogenous or exogenous [1, 3, 4, 5]. Patients typically present with acute‐onset, severe, colicky lower abdominal pain followed by sudden relief upon expulsion of the cast [3, 4]. This rare case provides definitive histopathological confirmation of an endometrial cast in an adult woman with PCOS on norethisterone, highlighting the importance of recognizing this underdiagnosed entity to prevent unnecessary surgical interventions.

## 2 Case History/Examination

A 27‐year‐old Syrian woman, Gravida 3, Para 2 (with two prior normal vaginal deliveries and one prior miscarriage, G3P2001, occurring approximately 3 years prior to this presentation), presented on the first day of menstruation with sudden‐onset severe colicky lower abdominal pain. Approximately 2 h later, she expelled a pinkish‐white fibrous mass per vagina, measuring 7 × 4.5 cm in size, soft, hemorrhagic, and polypoid in orientation, resulting in immediate resolution of pain. She reported minimal vaginal bleeding and denied nausea, vomiting, dizziness, or syncope. Her medical history was significant for untreated polycystic ovary syndrome diagnosed 3 years earlier. She had been taking continuous norethisterone 5 mg orally once daily, which she initiated to delay menstruation during Ramadan, for the preceding 2 months. She had no prior surgical history, and her family history was unremarkable. On examination, the patient was hemodynamically stable. Abdominal, cardiovascular, and respiratory examinations were within normal limits. On speculum examination, cervical ectropion was observed, and the cervix was dilated to approximately 1 cm, attributed to her multiparous external os. There was no adnexal tenderness or palpable masses. A urine pregnancy test was negative.

## 3 Differential Diagnosis, Investigations, and Treatment

**FIGURE 1:**
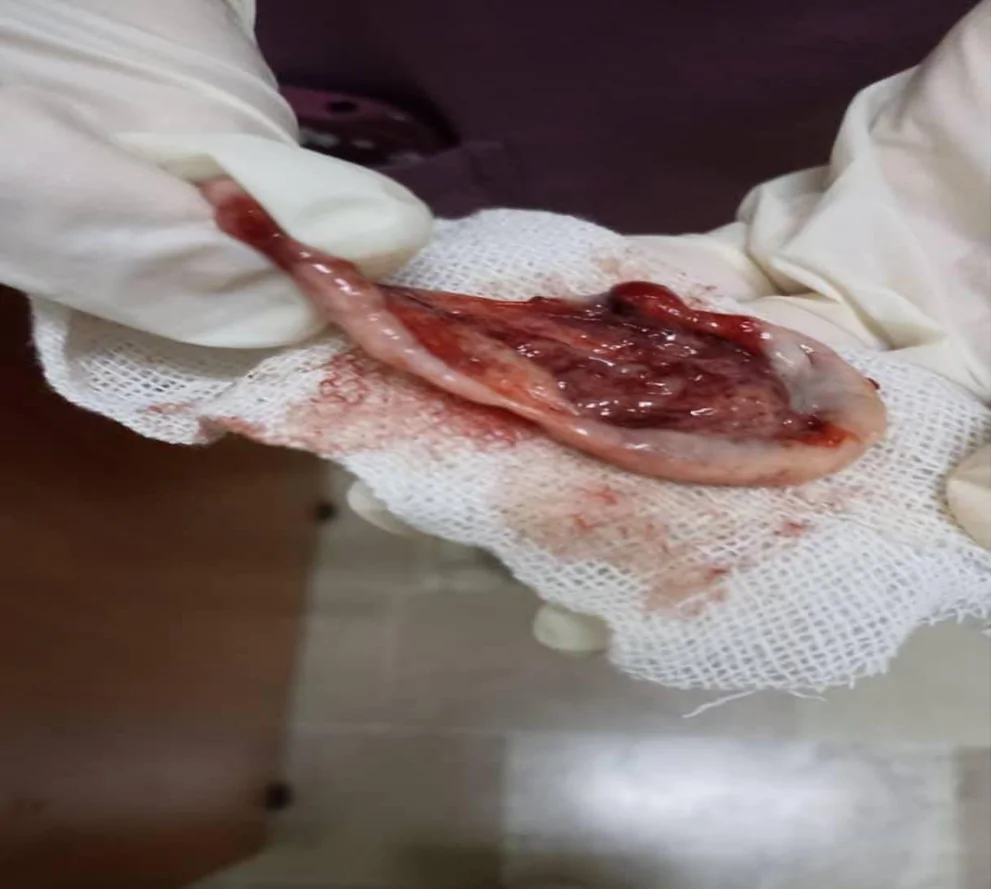
Gross photograph of the soft, hemorrhagic, polypoid decidual cast measuring 7 × 4.5 cm, expelled per vagina.

**FIGURE 2:**
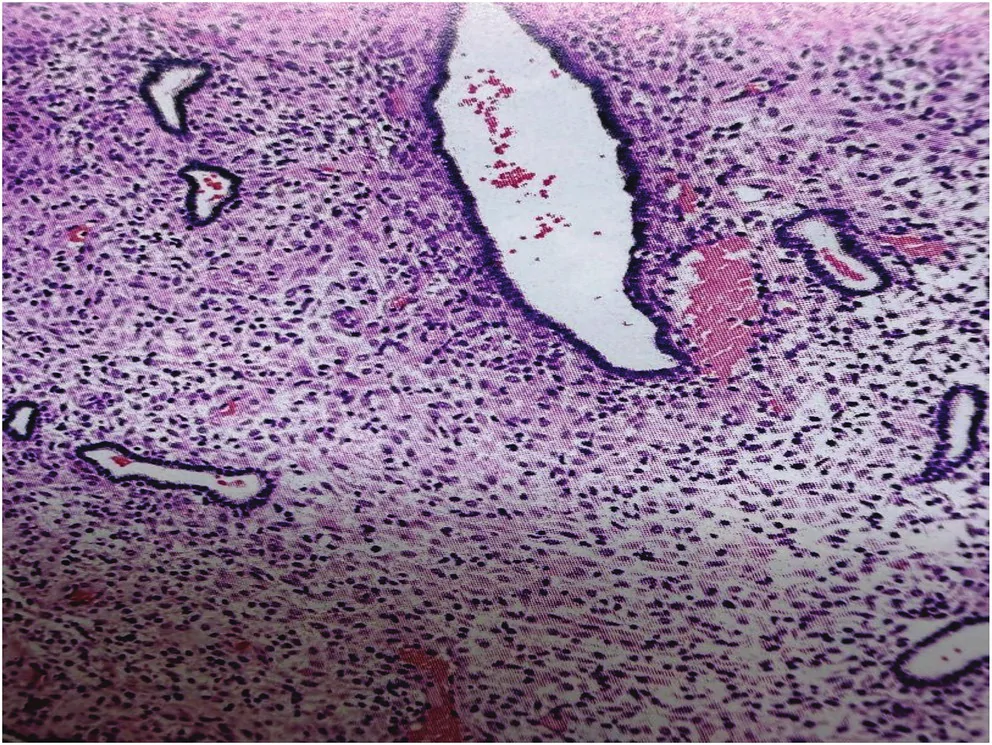
Hematoxylin and eosin stain showing pseudodecidualized endometrial stroma with inactive endometrial glands, consistent with exogenous progestin effect, without chorionic villi, atypia, or malignancy.

Given the acute presentation, differential diagnoses included early pregnancy loss, ectopic pregnancy, endometrial polyp expulsion, and retained products of conception. A urine pregnancy test was negative; serum quantitative beta‐hCG was not obtained as the point‐of‐care urine test was definitive and rapid quantitative testing was unavailable. Transabdominal pelvic ultrasound (TAS) was performed immediately upon the patient's arrival following cast expulsion (transvaginal ultrasound was unavailable due to equipment malfunction in the facility). Transabdominal pelvic ultrasound demonstrated a normal uterus with an endometrial line, bilateral ovaries showing the characteristic “string‐of‐pearls” appearance consistent with PCOS, and no free fluid in the pouch of Douglas. Gross examination of the expelled tissue revealed a soft, hemorrhagic, polypoid fragment measuring 7 × 4.5 cm (Figure 1). Histopathological analysis showed pseudodecidualized endometrial stroma with inactive glands consistent with progestin effect, without evidence of gestational tissue, atypia, or malignancy (Figure 2). Based on these findings, a diagnosis of decidual cast associated with continuous norethisterone use was established. Management was conservative; norethisterone was discontinued, and an antispasmodic agent (drotaverine hydrochloride) was prescribed for symptomatic relief.

## 4 Conclusions and Results (Outcome and Follow‐Up)

The patient experienced complete resolution of symptoms following expulsion of the decidual cast. No surgical intervention was required. At a three‐month follow‐up, she remained asymptomatic, with normal menstrual bleeding and no recurrence. This case underscores the importance of clinical recognition and histopathological confirmation of decidual cast to ensure appropriate conservative management and avoid unnecessary invasive procedures.

## 5 Discussion

Endometrial cast (membranous dysmenorrhea) is a rare gynecological entity characterized by the expulsion of the endometrial lining in its entirety, often maintaining the shape of the uterine cavity [1, 4, 5, 6]. The literature remains limited, with only a small number of reported cases, most of which occur in adolescents and are strongly associated with hormonal contraceptive use, particularly progesterone‐containing agents such as depot medroxyprogesterone acetate and combined oral contraceptives [1, 2]. The pathophysiology of endometrial cast formation remains incompletely understood and is considered multifactorial. Proposed mechanisms include progesterone‐induced decidualization, abnormal cell adhesion, spiral artery dysregulation, and an exaggerated endometrial response to hormonal stimuli [1, 2, 3]. A consistent finding across the literature is the central role of progesterone, either endogenous or exogenous, as a key contributing factor [1, 3]. Progesterone promotes endometrial decidualization and suppresses normal cyclical shedding, potentially resulting in en bloc sloughing of the endometrium [3]. However, recent evidence suggests that this phenomenon may not be purely dose‐dependent but rather related to an exaggerated or idiosyncratic endometrial response [2]. Most previously reported cases have occurred in adolescents, with 13 out of 21 documented cases in patients under 20 years of age [2], and fewer than 20 cases reported in adult women over the past two decades [2, 3]. In contrast, our case highlights that decidual cast can also occur in adult women, suggesting that additional factors beyond uterine immaturity contribute to disease pathogenesis [1, 4]. The passage of a decidual cast in this PCOS patient after just 2 months of continuous daily norethisterone therapy may be understood as an exaggerated and uncoordinated endometrial response occurring against the backdrop of a preexisting, dysfunctional uterine environment [7, 8]. Specifically, the endometrium in PCOS has been associated with progesterone resistance related to altered expression of progesterone receptor isoforms (PRA and PRB), with increased total PR expression in epithelial cells and impaired stromal responsiveness [8]. This is also associated with additional molecular aberrations, including hyperandrogenism‐driven disruption of WT1‐mediated decidualization, hyperinsulinemia‐induced activation of the PI3K/AKT‐NR4A1 pathway, and chronic low‐grade inflammation with elevated TNF‐α and IL‐6, which may collectively affect normal tissue adaptation and regenerative capacity [7, 8]. Therefore, when norethisterone was abruptly introduced into this already unstable environment, it may have contributed to a rapid and chaotic transformation of the endometrial cells rather than a smooth, cyclical maturation, potentially resulting in a structurally fragile tissue that lost its normal adherence and shed entirely as an intact cast [7, 8]. Most previously reported cases have been described in postmenarchal adolescents, with relatively few cases reported in adult women [1, 2]. This has led to the hypothesis that uterine immaturity, including incomplete development of uterine natural killer (uNK) cells and spiral artery regulation, may predispose younger patients to this condition [2]. In contrast, our case highlights that decidual cast can also occur in adult women, suggesting that additional factors beyond uterine immaturity may contribute to disease pathogenesis. In the context of the baseline sensitization and heightened uterine vulnerability associated with PCOS, our patient developed symptoms within an accelerated timeframe of just 2 months of continuous daily norethisterone therapy, compared to the standard average latency period of about 4.7 months seen in typical cases [2]. Clinically, endometrial cast expulsion is frequently misdiagnosed as miscarriage or ectopic pregnancy due to the acute onset of severe colicky abdominal pain and passage of tissue per vagina [1, 2, 4]. Consistent with previous reports, our patient experienced sudden severe pain followed by immediate relief after expulsion, which is a characteristic feature of this condition. From a diagnostic perspective, ultrasound findings, in conjunction with a negative pregnancy test and absence of free intraperitoneal fluid, are essential for excluding obstetric emergencies. Histopathological examination remains the gold standard for confirmation [4], revealing pseudodecidualized stroma without evidence of gestational tissue or malignancy. Regarding management, no standardized treatment guidelines currently exist. Most cases, including ours, are managed conservatively with reassurance and symptomatic treatment. While some authors recommend discontinuation of the offending hormonal agent due to its suspected etiological role [1], others report that continuation of hormonal contraception may be safe given the low recurrence rate [2].

## 6 Study Limitations

This case report has certain limitations. First, transvaginal ultrasound—the preferred imaging modality for detailed endometrial assessment—was unavailable due to technical constraints, requiring reliance on transabdominal imaging. Second, serum quantitative β‐hCG measurement could not be performed, relying instead on a high‐sensitivity point‐of‐care urine test.

## 7 Conclusion

Decidual cast should be considered in patients presenting with acute abdominal pain and passage of tissue, particularly in the context of progestin use. Awareness of this entity is essential to avoid misdiagnosis and unnecessary surgical intervention.

## Author Contributions

**Nagham Al‐hassoun:** data curation, formal analysis, investigation, resources, software, writing – review and editing, writing – original draft. **Mohammad Al Diab Al Azzawi:** validation, visualization, writing – original draft, writing – review and editing. **Hiba Abdullah:** validation, writing – original draft, writing – review and editing, project administration, resources. **Tuka Hamasho:** conceptualization, data curation, formal analysis, methodology, investigation, project administration, validation, visualization, writing – review and editing, writing – original draft. **Walid al saleh:** project administration, resources, supervision, validation, writing – review and editing. **Nazaha Al‐hassoun:** conceptualization, data curation, formal analysis, writing – review and editing, writing – original draft, software, resources, investigation.

## Funding

The authors have nothing to report.

## Consent

Written informed consent was obtained from the patient for publication of this case report and the accompanying clinical and histopathological images.

## Conflicts of Interest

The authors declare no conflicts of interest.

## Data Availability

Data sharing not applicable to this article as no datasets were generated or analysed during the current study.
